# Genetic characterization of Italian tomato varieties and their traceability in tomato food products-Sardaro-2012-Food Science & Nutrition-Wiley Online Library

**DOI:** 10.1002/fsn3.8

**Published:** 2013-01-08

**Authors:** Maria Luisa Savo Sardaro, Marta Marmiroli, Elena Maestri, Nelson Marmiroli

**Affiliations:** Department of Life Sciences, University of Parma, Parco Area delle Scienze11/A, 43124, Parma, Italy

**Keywords:** Food traceability, Solanum *lycopersicum L.*, SSRs molecular markers, tomato sauce

## Abstract

Genetic diversity underlies the improvement of crops by plant breeding. Landraces of tomato (*Solanum lycopersicum* L.) can contain valuable alleles not common in modern germplasms. The aim was to measure genetic diversity present in 47 most common tomato varieties grown in Italy, 35 were varieties used for processing and 12 were landraces considered “salad varieties”. Furthermore, we demonstrated the possibility that the variety traceability can be extended through the entire production chain. Diversity was measured using 11 microsatellite markers and 94 genotypes. Among the markers used, a total of 48 alleles were detected. A dendrogram based on total microsatellite polymorphism grouped 47 varieties into three major clusters at 0.75 similarity coefficient, differentiating the modern varieties from tomatoes landraces. The DNA markers developed confirmed the possibility to support the genotype identification all along the tomato production chain. The number of alleles and genotypes identified in the present work is the largest considering papers on food traceability.

## Introduction

Tomato (*Solanum lycopersicum* L.) is the most important fruit crop in the world; according to the FAOSTAT (Food and Agriculture Organization Statistics) 2010 report, the tomato market was 22 million tonnes/day in EU and 13 million tonnes/day in the USA (FAOSTAT 2010). Tomato was first introduced in Europe from Central and Southern America at the beginning of the 16th century and cultivated as an ornamental plant. In the 17th century, the species gained popularity because the fruits are an edible product and its cultivation spread rapidly throughout the Old World. This introduction resulted in a genetic bottleneck, narrowing the genetic diversity of the cultivated germplasm in Europe (Rick 1976). In Europe, tomato plants have been most successful in the Mediterranean countries, including Spain and Italy (Soressi 1969; García-Martínez et al. 2006) In these countries, *S. lycopersicum* found a secondary centre for diversification, which resulted in a wide array of variations including round, obovoid, long, heart, rectangular, and even bell pepper–shaped fruits (Bailey et al. 1960). All these variations are still present among the tomato landraces used for fresh consumption, the so-called “salad tomatoes” that are widely grown in both Spain and Italy (García-Martínez et al. 2006; Acciarri et al. 2007; Mazzucato et al. 2008).Tomato breeding projects have improved characteristics such as disease resistance, fruit abscission, soluble solids, fruit size, texture, flavor, pigmentation, and storage ability. The most commercial varieties of tomato for industrial transformation are F1 hybrid.

Approximately 350 varieties are registered at the Italian National Register of Varieties, and 65 of them are considered traditional varieties (http://www.sementi.it). A variety is identified by a set of morphological characteristics according to the UPOV (International Union for the protection of new varieties of Plants). In Italy in 2010, more than 0.6 million tons of tomato varieties for processing were produced and 0.06 million tons of salad tomatoes cultivars were harvested.

As tomatoes are eaten directly raw or added to other food items, a variety of processed products such as paste, whole peeled tomatoes, diced products, and various forms of juice, sauces, and soups have gained significant acceptance. Considering that there are more varieties of tomato sold worldwide than any other vegetable, the strategic development of a food-chain approach to trace food quality and safety must be considered within the global context that is constantly evolving in terms of normative requirements. Nowadays, food characterization is a challenging topic, which goes alongside with raw matter traceability because it includes both authenticity and geographical origin determination. In particular, internal traceability has been indicated as a production action to improve reliability of labeling, to certify the origin and the quality of products on the market, and to prevent fraudulent or deceptive labeling (European Commission 2002). The European Union has considered the use of high-quality raw material in food production as a prerequisite to obtain genuine and safe products of adequate nutritional value (White Paper on Food Safety COM/99/719). Consequently, internal traceability is assuming a particular relevance in the worldwide process of global traceability.

Methodologies based on genetic and molecular biology are acquiring great interest for their applicability to track a given item at any stage along the food supply chain, “from farm to fork” (Di Bernardo et al. 2005). The approach based on these techniques is known as “food genomics”. One of the most important tools in this context is the polymerase chain reaction (PCR), which allows the identification of traces of genomic DNA that may residue in a food matrix from the principal component and/or from contaminants (Marmiroli et al. 2003, 2009; Agrimonti et al. 2011). Morphological descriptors do not always allow the quantification of genotypic difference, because quantitative characters can be altered by environmental factors (Cooke 1995). In contrast, molecular markers such as restriction fragment length polymorphism (RFLP), random amplified polymorphic DNA (RAPD), amplified fragment length polymorphism (AFLP), single nucleotide polymorphism (SNP), and simple sequence repeats (SSR) can provide an effective tool for variety identification as they are independent of environmental effects (Lee and Henry 2001; Sim et al. 2009). Among the different available marker systems, SSR markers have become important for variety identification because of their property of genetic codominance, high reproducibility, and multiallelic variation (Powell et al. 1996). The work of Smulders et al. (1997), Bredemeijer et al. (2002), He et al. (2003), Frary et al. (2005), García-Martínez et al. (2006), Song et al. (2006), Kwon et al. (2009), Turci et al. 2010, and Caramante et al. (2011) confirmed the utility of DNA molecular markers for studying genetic diversity and variability in the genus *Solanum* and for selecting tomato cultivars. SSRs are better performing for identification of varieties because they are codominant markers, while SNP, AFLP, RAPD, and other methodologies are only able to highlight the dominant alleles. In comparison to the other codominant technique RFLP, SSR experiments are faster to perform and the results are more clear cut.

The aims of this work were mainly the genetic characterization of the more popular Italian tomatoes cultivars both for fresh market salad tomatoes and for industrial processing using DNA methods and SSRs, and their traceability along the entire tomato food chain.

The extent of this study goes beyond the range of Italian market and the interests of Italian consumers, because salad tomatoes are sold all over Europe and canned tomatoes enter the world global market. For example, the cultivars Perfect Peel and Guadalete are the most widespread in Europe for tomatoes processing (Grolier et al. 2001).

DNA fingerprinting provides a suitable tool to track and trace the tomato supply chain “from farm to fork”, ensuring not only authenticity and integrity of the products but also the absence of any possible genetic contamination by other species or unwanted components (Marmiroli et al. 2003, 2009; Agrimonti et al. 2011).

## Materials and Methods

### Plant materials and food matrices

Forty-seven tomato (*Solanum lycopersicum* L.) varieties, more represented in 2009 in the Italian seed market, are reported in Table 1. We analyzed three monovarietal tomato sauces (HEINZ 3406 (H), Perfect Peel (PP), Pata Rojal (PR)), and one mixed tomato sauce (PP + PR + H) and the relative seeds and fresh tomato fruits (Table 2). For each lot, we isolated DNA from three individuals, which in all instances, displayed the same allelic profile; the allelic profile is all the possible distinct versions of the gene that vary in DNA sequence for the same locus(Buchanan et al. 2000). The tomato sauces were produced in a small-scale processing plant in collaboration with the Experimental Station for the Food Preserving Industry in Parma (SSICA) following the same procedures used in the large-scale industrial process.

**Table 1:** List of recent research papers on correctly and incorrectly identified *Rubus coreanus* fruit based on anthocyanin profile shown in Figure 2.

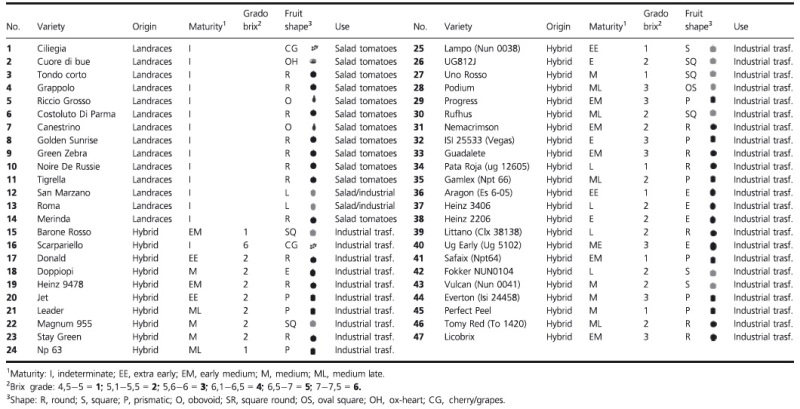

**Table 2:** Tomatoes varieties utilized for samples production (sauce, seeds, and fruits) used in the experiment

Varieties	Type of sample	Abbreviations	Extraction method
Perfect Peel (PP)	Tomato sauce	PerfectPeeltomsau	Wizard Protocol
Pata Rojal (PR)	Tomato sauce	PataRojaltomsau	Wizard Protocol
Heinz 3406 (H)	Tomato sauce	Heinz3406tomsau	Wizard Protocol
Mix (PP+PR+H)	Tomato sauce	Mix (PP+PR+H)	Wizard Protocol
Perfect Peel	Seeds	PerfectPeelseed	Genelute Plant Genomic Kit
Pata Rojal	Seeds	PataRojalseed	Genelute Plant Genomic Kit
Heinz 3406	Seeds	Heinz3406seed	Genelute Plant Genomic Kit
Perfect Peel	Fresh tomatoes	PerfectPeelfruit	Wizard Protocol
Pata Rojal	Fresh tomatoes	PataRojalfruit	Wizard Protocol
Heinz 3406	Fresh tomatoes	Heinz3406fruit	Wizard Protocol

## DNA isolation

Genomic DNA was isolated from 100 mg of frozen young leaves and seeds or 300 mg of fruits or tomato sauce. Plant organs were ground to fine powder with liquid nitrogen and DNA isolation was performed following the procedure of the “Genelute Plant Genomic DNA Kit” (Sigma-Aldrich, St. Louis, MO). Frozen fruits and tomato sauce were processed using Wizard Protocol (Zimmermann et al. 1998; Turci et al. 2010). This kit, designed for food matrices, combines the use of prepacked columns and reagents prepared by the user. The procedure was performed according to the manufacturer's instructions as described by Zimmermann et al. (1998). In order to obtain statistically suitable data, three samples representative of different plants, for each variety, were analyzed. For the tomato sauce production, 100 kg of tomato were collected from each variety analyzed. DNA concentration and purity were determined using a Cary 50 Spectrophotometer (Varian Inc., Torino, Italy). The DNA extracted from all samples was tested for PCR amplificability using primers targeting a single-copy gene LAT 52 (GenBank accession number P13447)(Yang et al. 2005).

## PCR amplification

### Test for DNA amplificability and SSR analysis

DNA extracted from tomato samples listed in Table 2 were tested for PCR amplificability with the LAT 52 gene (GenBank accession number P13447) (Yang et al. 2005). PCR amplificability of DNA isolated from processed food samples was tested using the following primers, Lat1 For AGACCACGAGAACGATATTTGC and Lat2 Rev TTCTTGCCTTTTCATATCCAGACA. All PCR reactions were carried out using a Veriti™ Thermal Cycler (Applied Biosystems, Foster City, CA). PCR assays were performed in a final volume of 25 *μ*L of PCR amplification reaction mixture containing 1 U of Go *Taq* DNA Polymerase (Promega, Madison, WI), 0.5 *μ*L 10 mm dNTP mix, 0.25 *μ*mol/L of each primer, forward and reverse primer (Sigma-Aldrich), 1× Go *Taq* reaction buffer (Promega, Madison, WI), and 30 ng of genomic DNA. Amplification reactions were run under the following conditions: DNA denaturation at 94°C for 2 min, followed by 40 cycles with: denaturation at 94°C for 45 s; annealing at 60°C for 45 s according to the melting temperature of the primers; extension at 72°C for 45 s, and at the end, a primer thermal extension at 72°C for 7 min.

**Figure 1:**
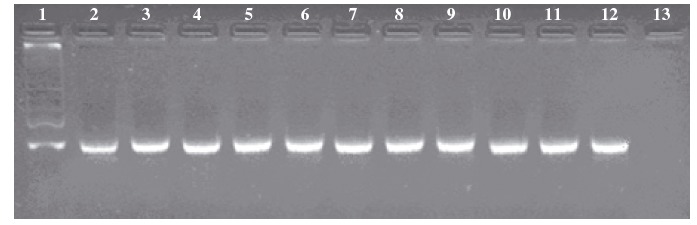
Test for the amplificability of DNA isolated from sauces, seeds, and tomato fruits listed in Table 2. Agarose gel electrophoresis of LAT52 PCR products. 1: 100 bp molecular marker; 2,3,4: Perfect Peel, Pata Rojal, and Heinz 3406 Tomatoes sauce; 5: Tomato sauce of the Mix (PP+PR+H); 6,7,8: Perfect Peel, Pata Rojal, and Heinz 3406 seeds; 9,10,11: Perfect Peel, Pata Rojal, and Heinz 3406 fresh tomatoes, respectively; 12: positive control Heinz 3406 leaves; 13: negative control.

Following amplification, 10 *μ*L of the PCR products were analyzed by electrophoresis on 3% agarose gels run with 1×TBE at 80 V (89 mmol/L Tris borate, 89 mmol/L boric acid, 2 mmol/L EDTA) (Sambrook et al. 1989). The gels were stained with ethidium bromide, visualization and acquisition of digital images using Bio-Rad Gel Doc 2000 instrument with Proprietary Software (Bio-Rad, Hercules, CA; see Fig. 1).

The Simple Sequence Repeats (SSRs) analysis on the different cultivars of fresh tomatoes, enlisted in Table 1, and on processed tomatoes shown in Table 2, was performed using a combination of 11 tomato-specific SSR primers (Table 3) (Suliman-Pollatschek et al. 2002; He et al. 2003). One of each primer pair was 5‵ labeled with the IRD700 dye (Eurofins MWG-Operon, Ebersberg, Germany). PCR assays were performed in a final volume of 25 *μ*L containing: 1 U of Go *Taq* DNA Polymerase (Promega), 0.5 *μ*L 10mm dNTP mix, 0.25 μmol/L of each primer, 1× Go *Taq* reaction buffer (Promega, Madison, WI), and 100 ng of genomic DNA. Amplification reactions were performed as previously described.

**Table 3:** SSR loci used in this study and their main parameters

SSR name	Chr. location	Core motif	Reference	Observed size range (bp)	Allele no.	PIC	Ta (°C)
LE at002	–	(AT)9	He et al. (2003)	201–205	3	0,56	59
LE aat002	–	(AAT)12	He et al. (2003)	99–104	3	0,49	63
LE ga003	–	(GA)20	He et al. (2003)	231–235	3	0,54	59
LE tat002	–	(TAT)12	He et al. (2003)	195–201	3	0,51	59
LE aat007	–	(AAT)12	He et al. (2003)	93–99	3	0,53	59
SSR248	10	(AT) 21	Solanaceae Genomics Network [http://www.sgn.cornell.edu]	241–252	7	0,78	57
SSR47	6	(AT) 14	Solanaceae Genomics Network [http://www.sgn.cornell.edu]	189–201	4	0,42	56
SSR603	4	(GAA) 8	Solanaceae Genomics Network [http://www.sgn.cornell.edu]	235–254	6	0,35	58
TOM236	9	(AT) 16	Suliman-Pollatschek et al. (2002)	156–211	9	0,59	56
SSR70	9	(AT) 20	Solanaceae Genomics Network [www.sgn.cornell.edu]	115–121	3	0,23	59
TOM210	4	(ATA) 15	Suliman-Pollatschek et al. (2002)	218–224	4	0,59	56

SSR, simple sequence repeats, Chr., chromosome; PIC, polymorphic information content.

To verify the presence of amplified DNA fragments, the PCR products were first separated by agarose gel electrophoresis using a 3% (w/v) agarose gel in TAE 1×, stained with 1 *μ*g/mL of ethidium bromide solution and visualized using a Bio-Rad Gel Doc 2000 (Bio-Rad) (Sambrook et al. 1989). The allelic identification was performed using a CEQ 2000 gene analysis system (Beckman Coulter, High Wycombe, U.K.). The sizes of all the alleles were identified using automated fragment analysis and a CEQ DNA size standard 400 (Beckman Coulter).

## Data analysis

For each SSR locus, we calculated the number of alleles and the polymorphic information content (PIC) (equivalent to the expected heterozygosity, He):


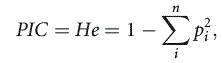


*n*, *i*,∊ N, *p* ∊ R+, where *p*_*i*_ is the frequency of the *i*^th^ allele among all the *n* alleles possible at a given locus (Hartl and Clark 1997). Calculations were performed using the GenAlEx 6.2 software (Peakall and Smouse 2006, free software distributed by the authors) and presented in Table 3. For cluster analysis, monomorphic SSR loci were excluded. The estimation of the genetic similarity between all the varieties was calculated according to Nei and Li (1979)*,* and the corresponding phylogenetic trees were drawn with the unweighted pair group method using arithmetic average (UPGMA) clustering method through the GDA software Version 1.0 (d16c) (Genetic Data Analysis) (free software distributed by the authors; Sneath and Sokal 1973; Lewis and Zaykin 2001).

## Results and Discussion

### DNA isolation from fresh tomato, processed tomato, and test for amplificability

The DNA extraction performed on leaf, seeds, and fresh tomatoes gave the expected results in term of quality and quantity (70 ± 30 ng of DNA per mg of tissue). The DNA isolation from processed tomatoes provided yields and results predictably quite variably because of the low amount of DNA present in the processed materials and its high degree of degradation (Bauer et al. 2003; Turci et al. 2010). Our previous evaluation of DNA-isolation procedures from tomato matrices indicated that a commercial kit, Wizard (Promega), provided the best performance (Turci et al. 2010). The kit was used for the DNA extraction from four tomato sauce types. Both amount and purity of DNA were examined spectrophotometrically (average: 0.8 ± 0.2 ng of DNA per mg of tissue). The DNA extracted from tomato sauces were then tested for PCR amplificability using primers targeting a single-copy gene LAT 52 (GenBank accession number P13447) (Fig. 1; Yang et al. 2005).

### SSRs analysis

Eleven SSR loci representatives of various repeat classes were the best performers in terms of DNA amplification among a pool of 20 primers, they were also selected for their high polymorphism and their ability to generate small PCR products. All of them were described in publications or on the website of Solanaceae Genomics Network (Table 3) and were used to genotype 47 different tomato cultivars and four tomato sauce types, plus the corresponding seeds and tomato fruits. The number of alleles identified by each marker ranged from three to nine with a mean of 4.36. Size differences between the smallest and largest alleles varied from 93 bp to 254 bp. Polymorphism information content ranged from 0.23 to 0.78, with an average of 0.508 (Table 3). Each cultivar was characterized by a specific allelic profile and, therefore, could be discriminated from all the others (Table 4). DNA samples extracted from processed tomatoes were analyzed using 8 out of 11 SSR loci according to their amplification performance, and it was possible to identify each variety and discriminate them even when they were mixed (Table 4). The most consistent results for traceability of processed tomatoes were obtained by using low amounts of DNA template (from 10 to 25 ng) and by using SSR markers that amplified products with a size up to 200 bp. There were only few exceptions longer than 200 bp which showed a good amplification efficiency, like the 235 bp of Lega003 (Table 4). Few among the 11 SSR markers selected were not able to give a good amplification on the processed materials. This was attributed by other authors to the high level of DNA degradation occurring during the industrial process (Caramante et al. 2011). This interpretation is only partially comprehensive of the results obtained. In fact, alleles above 200 bp (like SSR248, SSR603, TOM210, Lega003) can be detectable (Lega003) or not (SSR248, SSR603, TOM210) independently of their size. Therefore, the step of the amplification which is at the basis of detectability showed a weakness in the detection.

**Table 4:** Alleles profiles of seeds, leaves, fruits, and sauces of the three tomato varieties used in the traceability study by using eight SSR markers

	Letat002		LEat002		LEaat002		LEga003		LEaat007		SSR 47		TOM236		SSR70	
Seeds																
HEINZ 3406	195	195	201	201	104	104	231	233	99	99	189	189	173	175	119	119
PATA ROJAL	195	198	201	205	101	101	235	235	96	99	189	201	173	175	119	119
PERFECT PEEL	195	198	201	205	101	104	235	235	96	99	189	189	173	175	119	119
Leaves																
HEINZ 3406	195	195	201	201	104	104	231	233	99	99	189	189	173	175	119	119
PATA ROJAL	195	198	201	201	101	104	235	235	96	99	189	201	173	175	119	119
PERFECT PEEL	195	198	201	205	101	104	235	235	96	99	189	189	173	175	119	119
Fruits																
HEINZ 3406	195	195	201	201	104	104	231	233	99	99	189	189	173	175	119	119
PATA ROJAL	195	198	201	201	101	104	235	235	96	99	189	201	173	175	119	119
PERFECT PEEL	195	198	201	205	101	104	235	235	96	99	189	189	173	175	119	119
Sauces																
HEINZ 3406	195	195	201	201	104	104	233	235	99	99	189	189	173	175	119	119
PERFERCT PEEL	195	198	201	205	101	104	231	233	96	99	189	189	173	175	119	119
PATA ROJAL	195	198	201	205	101	104	233	235	96	99	189	201	173	175	119	119
MIX (PP+PR+H)	195	198	201	205	101	104	231/233	235	96	99	189	201	173	175	119	119

The estimation of varieties specific alleles gave results mainly on very old varieties of salad tomatoes (Table 5) demonstrating the effectiveness of the conservation of these varieties, in particular on the cultivar San Marzano, that is considered a PDO (Protected Designation of Origin) variety. Moreover, it was possible to find a specific allele (156 bp) for Scarpariello variety at the locus TOM236 present only in another cultivar, the Ciliegia, both highly similar in the size and the morphologic characteristics of the berries (Table 5).

**Table 5:** Specific alleles present in the set of populations used in the study

Locus	Allele	Frequency	Found in
SSR248	252	0.50	Costoluto di Parma
SSR248	250	0.50	Costoluto di Parma
SSR47	199	0.50	Leader
SSR603	235	0.50	Tigrella
SSR603	242	0.50	Roma
SSR603	248	0.50	Podium
TOM236	211	0.50	Noire de Russie
TOM236	185	0.50	Cuore di bue
TOM236	207	0.50	Gamlex
TOM210	224	1.0	San Marzano

**Figure 2:**
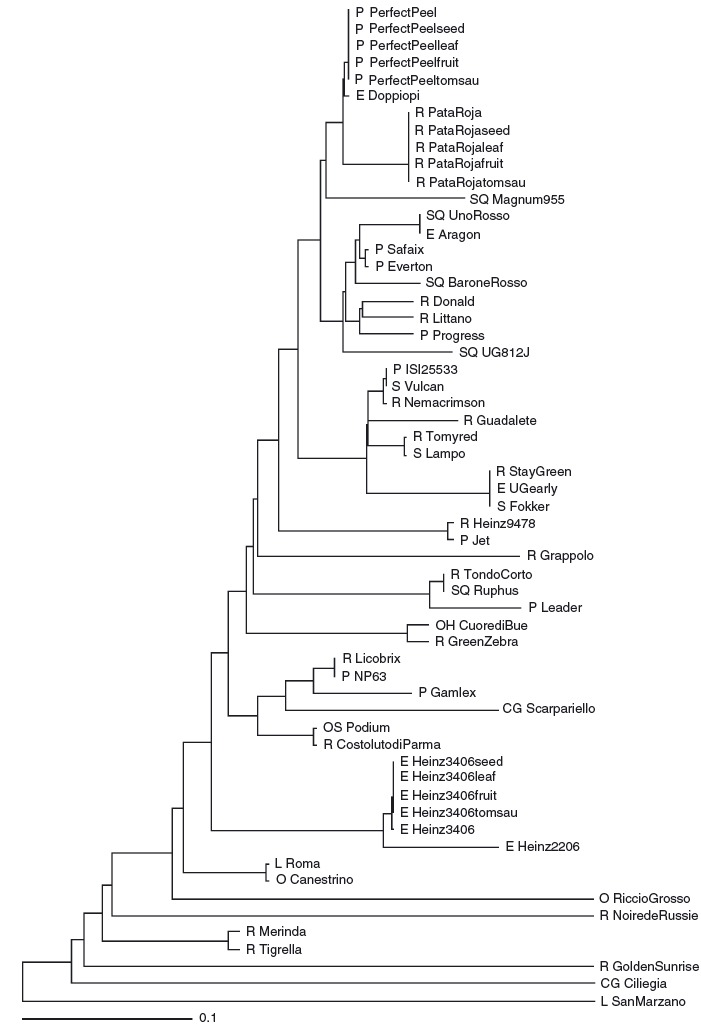
Hierarchical clustering (UPGMA algorithm) of 47 varieties, four tomatoes sauces, seeds, and fruits based on Nei genetic identity calculated using SSRs data. The fruit shape is labeled: R, round; S, square; P, prismatic; O, obovoid; SR, square round; OS, oval square; OH, ox-heart; CG, cherry/grapes; E, ellipsoid.

Figure 2 represents through a UPGMA hierarchical clustering, the grouping of different cultivars through a phylogram picture; different levels of discrimination were achieved among the cultivars according to their allelic profiles, for example the “salad tomato cultivars” are discriminated from the processing tomato, and within their group they are further subdivided. In fact, the phylogenetic tree based on total microsatellite polymorphism grouped the 47 varieties into two major clusters, differentiating first of all the modern varieties from tomatoes landraces, which are the more ancient cultivars, whereas the modern varieties for processing were grouped in one main cluster (Fig. 2).

Considering specifically the three varieties used for the sauces production and used in the analysis extracted from seed, leaf, fruit, and sauce, the method demonstrated the possibility to discriminate and follow up all along the tomato food chain. In particular, the phylogenetic tree gave a support for their discrimination potentially in any tomato-based matrices. Moreover, in Table 4 is shown the possibility of identifying the processed varieties present in a mixed tomato sauce according to the list of alleles present in leaf and seed.

The results indicate that despite the limited genetic diversity present in commercial tomato varieties, DNA fingerprinting based on SSRs is able to discriminate varieties which are morphologically similar and genetically close, and can therefore be used for the identification and protection of genetically valuable materials. The results obtained are particularly reliable because the experiments were performed on a wide group of varieties (both industrial and landraces) and types of samples along the whole tomato production chain (seeds, leaves, fruits, and processed tomato sauce). In this study, the processed products are all genetically certified and homogeneous and reproducible processing methodologies were used in order to follow the genetic fingerprint of each cultivar.

Moreover, the SSR markers analysis confirmed the possibility to assist the genotype identification all along the tomato production chain, providing the factual possibility of tracking tomato cultivars in the processed tomato products by using DNA technology and molecular markers. For example, it is feasible to trace processed tomato for authenticity and identity preservation, and as a tool for tomato industry to improve breeding programs, quality control, and internal traceability. However, to obtain an effective implementation of the traceability system of tomato plants and products and a high level of varieties discrimination, it is necessary to achieve the fingerprinting of all the commercial varieties and develop a comprehensive DNA database of tomato cultivars. Considering the increasing world demand for processed tomato products and market globalization, our data support the promotion of an integrated approach within the food processing industry to identify key premium products, hopefully adding value and strength to competing brands in the agri-food market.

Food genomics constitutes a fundamental tool in assessing the quality of fresh and processed tomato products. In particular, within the mooted topic of products labeling, genetic analysis conveys an indispensable suit of methodologies to ensure the veracity and reliability of the system. Moreover, in this work, we have demonstrated the efficacy of the SSRs molecular markers in the identification and discrimination of cultivars, even within processed food, relying on their codominance and specificity.
